# Overcoming Resistance to Standard-of-Care Therapies for Head and Neck Squamous Cell Carcinomas

**DOI:** 10.3390/cells13121018

**Published:** 2024-06-11

**Authors:** Chester Gauss, Logan D. Stone, Mehrnoosh Ghafouri, Daniel Quan, Jared Johnson, Andrew M. Fribley, Hope M. Amm

**Affiliations:** 1 Carman and Ann Adams Department of Pediatrics, School of Medicine, Wayne State University, Detroit, MI 48202, USA; chester.gauss@wmed.edu (C.G.); mehrnoosh.ghafouri@med.wayne.edu (M.G.);; 2 Oral & Maxillofacial Surgery, School of Dentistry, University of Alabama at Birmingham, Birmingham, AL 35294, USA; ldstone@uab.edu; 3 Department of Otolaryngology Head and Neck Surgery, School of Medicine, Wayne State University, Detroit, MI 48202, USA; daniel.quan@wayne.edu (D.Q.);; 4 Molecular Therapeutics Program, Karmanos Cancer Institute, Wayne State University, Detroit, MI 48202, USA

**Keywords:** HNSCC, oral cancer, cisplatin, cetuximab, chemotherapy resistance, clinical trials

## Abstract

Although there have been some advances during in recent decades, the treatment of head and neck squamous cell carcinoma (HNSCC) remains challenging. Resistance is a major issue for various treatments that are used, including both the conventional standards of care (radiotherapy and platinum-based chemotherapy) and the newer EGFR and checkpoint inhibitors. In fact, all the non-surgical treatments currently used for HNSCC are associated with intrinsic and/or acquired resistance. Herein, we explore the cellular mechanisms of resistance reported in HNSCC, including those related to epigenetic factors, DNA repair defects, and several signaling pathways. This article discusses these mechanisms and possible approaches that can be used to target different pathways to sensitize HNSCC to the existing treatments, obtain better responses to new agents, and ultimately improve the patient outcomes.

## 1. Introduction

Head and neck squamous cell carcinomas (HNSCC) constitute the sixth most common type of cancer worldwide, with an incidence of 630,000 cases and more than 350,000 deaths per year [1]. HNSCC are classified based on the site of tumor origin and vary in terms of their etiology, prognosis, and genomic alterations. Oral and oropharyngeal cancers (OSCC) are some of the most common HNSCC. The worldwide incidence of OSCC is estimated to be more than 300,000 people per year, with 145,400 estimated deaths per year. Reported overall five-year survival rates range from 50 to 60%, with recurrence expected in 32–50% of patients after initial treatment [2,3,4]. Both the incidence and mortality rates are higher in men than in women, which has been attributed to the higher rates of tobacco and alcohol use by men [5,6,7]. Notably, the 5-year survival rate for OSCC in Black men (30.8%) is much lower than that in Caucasian (53.6%), Asian (57.9%), and Hispanic (53.8%) men in the U.S. [7]. However, the incidence and mortality rates have continued to gradually increase in both men and women and in all races since 2008. 

The current standard of care for HNSCC, including OSCC, is surgical resection. If the disease is caught early, resection can lead to good cosmetic and survival outcomes. Although it has been suggested that OSCC follows a progression from atypia to dysplasia to full invasive malignancy, most cases of the disease are discovered when it is already in an advanced stage [8]. Surgery in these cases often leads to significant disfigurement and comorbidities such as issues with eating and swallowing. Surgery may require extensive reconstructions, as well as specialized postoperative care [2]. Due to the advanced stage at discovery, these cancers are also typically treated with adjuvant radiotherapy and/or chemotherapy, which lead to further comorbidities.

## 2. Non-Surgical Treatments for HNSCC

Radiation has been used to treat HNSCC for almost a century, and continues to be a mainstay of treatment [9]. It can reduce the need for extensive surgical resection, preserving the function of the tissue and allowing for better cosmetic outcomes. However, while there have been considerable improvements in the dosing and administration of radiation over the years, such as the introduction of intensity-modulated radiotherapy and brachytherapy, radiotherapy is still associated with adverse effects, including tissue and bone necrosis [10]. Many patients also relapse following radiotherapy, requiring re-irradiation, surgery, or chemotherapy [11]. In addition, radiation is not applicable for all HNSCC tumors, with the tumor site being a major factor determining the optimal treatment.

Chemotherapy, particularly with platinum-based agents, has been the preferred pharmacological treatment for individuals with HNSCC in North America and Europe [12]. A cisplatin analog, carboplatin, along with paclitaxel and 5-fluoruracil (5-FU), may be prescribed for the treatment of OSCC and other HNSCC tumors as part of several different regimens approved within the guidelines set forth by the National Comprehensive Cancer Network (NCCN) [13]. Although patients often have a favorable initial response, most of them later develop resistance [14]. In addition, the main chemotherapeutic agents used to treat HNSCC, such as cisplatin, are non-selective and associated with adverse side effects [15,16].

**Figure 1:**
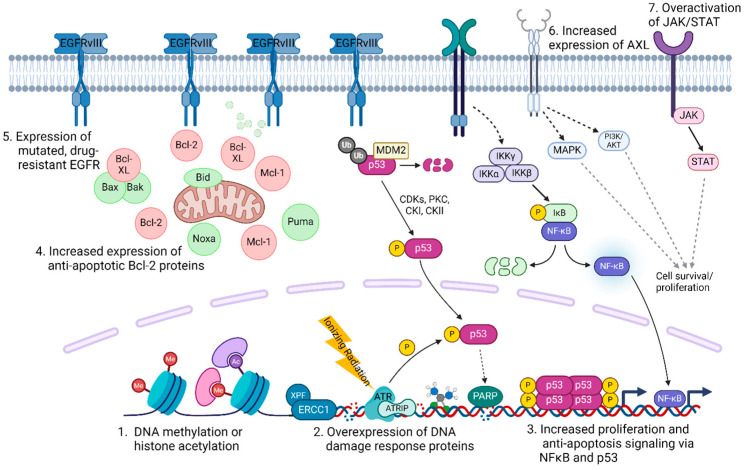
Mechanisms of resistance to standard-of-care therapies for HNSCC. Created with BioRender.com.

For the past few decades, the only advance in pharmacological treatment was the introduction of epidermal growth factor receptor (EGFR) inhibitors [17]. Unfortunately, many patients are innately unresponsive to these agents, and most of those who are initially sensitive develop resistance during treatment [17,18]. More recently, immune checkpoint inhibitors, including pembrolizumab and nivolumab, were approved for HNSCC therapy, but only about 20% of patients respond to these agents [19,20]. In this review, we describe several of the signaling pathways and genetic mutations that lead to the resistance of HNSCC to various treatments (Figure 1) and discuss possible approaches to subvert this resistance. This review has the most up-to-date and comprehensive information on the mechanisms of cisplatin and cetuximab resistance and clinical trials attempting to overcome this therapeutic resistance.

## 3. Major Pathways Involved in the Resistance to Treatment

### 3.1. Histone Acetylation and Epigenetics

Epigenetic alterations are changes in the chemical structure of DNA that affect transcription but do not change the coding sequence (e.g., methylation or de-methylation on cytokines that precede a guanine residue). It is well established that epigenetic alterations are involved in the development of many cancers, including HNSCC, and can modulate the resistance to chemotherapeutic agents and radiation [21,22,23,24,25]. Several epigenetic mechanisms underlie the development of treatment resistance, such as DNA methylation, chromatin remodeling, and histone acetylation [22,26,27].

The nicotinamide N-methyltransferase (NNMT) influences epigenetics, carcinogenesis, and the response to treatment by regulating several molecules, including histone deacetylases [28]. The protein is reported to be involved in processes as diverse as drug metabolism and DNA repair, cancer cell stemness, and autophagy [29]. Given its role in DNA methylation and energy metabolism, it is not surprising that NNMT upregulation has been observed in various cancers, including HNSCC, and its overexpression is associated with more aggressive behavior and resistance to chemo- and radiotherapy [30].

### 3.2. Defects in DNA Repair

Cisplatin exerts its cytotoxic effects through the formation of DNA-platinum adducts that lead to double-strand breaks (DSB), resulting in genomic instability and subsequent cell death [12]. Nucleotide excision repair (NER) has been shown to allow cancer cells to repair the cisplatin damage before it can cause cell death [31]. The resistance in many cancers has been linked to increased expression of excision repair crosscomplementing-1 (ERCC1), a key protein involved in NER. In support of this, high ERCC1 expression is associated with a significantly worse progression-free survival (PFS) and overall survival (OS) in patients with HNSCC, including those treated with cisplatin [12,32,33,34]. ERCC1 overexpression may serve as a predictive biomarker for resistance and may be tested prior to initiating therapy [31,33]. The repair of DSBs can also be mediated by ataxia telangiectasia and Rad3-related (ATR), which activates checkpoint kinase 1 (Chk1), leading to cell cycle arrest and repair of the affected DNA. ATR is involved in the response of cancer cells to both chemotherapy and ionizing radiation [35].

The most well-studied gene involved in carcinogenesis and the response to therapy is *TP53*. The protein product of the gene, p53, has been described as the “guardian of the genome”, and p53 mutations are thought to be involved in about half of all cancers [36,37,38]. Numerous studies have shown that mutations in *TP53* are associated with resistance to various anti-cancer treatments and a poorer prognosis [39,40,41]. Interestingly, patients with human-papilla-virus-positive (HPV+) HNSCC show higher treatment responses and better survival rates than those who are negative. This may be because HPV+ patients have low rates of *TP53* mutation, likely because HPV’s interaction with wild-type p53 is integral to its proliferation [42]. The wild-type p53 present in HPV+ cases may aid in the response to chemotherapy by regulating proteins involved in the DNA damage response (DDR). E1 is a viral protein produced in HPV-infected cells that activates both ATR and ATM, which activate the DDR. However, the activated DDR machinery is used to replicate the virus, making it unavailable to repair damage produced by cisplatin, leading to higher chemosensitivity [43].

In contrast, 75–85% of HPV-negative (HPV-) patients have mutated *TP53* [11,43]. In a study of 510 HNSCC patients (the majority had oral or oropharyngeal cancer), 70.4% of patients had a *TP53* mutation, and these patients had a poorer OS rates than patients with the wild type gene [44]. Patients with mutant *TP53* have also been shown to have a reduced response to platinum-based chemotherapeutic regimens. In a study of 53 OSCC patients, 45% of patients had mutated *TP53* and they had lower response rates to treatment with cisplatin + 5-fluorouracil [45]. Lindermann et al. stratified *TP53* mutations as low- and high-risk [46]. The high-risk *TP53* mutant group was 10-fold more likely to have residual disease after treatment with cisplatin-based chemotherapy than the low-risk group. The p53 protein has also been shown to be involved in the response of malignant and normal cells to a wide variety of other anti-cancer treatments, ranging from radiation to immunotherapy [47,48].

### 3.3. BCL-2 Signaling and Apoptosis

Increased expression of some members of the BCL-2 family of proteins is another mechanism which HNSCC relies on to evade apoptosis and induce chemo- and radioresistance [15]. BCL-2, BCL-XL, and MCL-1 are all pro-survival members of this family that inhibit the pro-apoptotic features of other family members, such as BAX and BAK [16,49,50,51]. The pro-apoptotic members of this family induce apoptosis by increasing the permeability of mitochondrial membranes. The increased permeability results from the insertion of pore complexes that release reactive oxygen species and cytochrome c. High concentrations of these molecules activate the caspase cascade, ultimately leading to programmed cell death [16,49,52]. In some HNSCC, the delicate balance between the pro-apoptotic and pro-survival proteins is disrupted, leading to an increased tumor burden and resistance to treatment.

### 3.4. Other Signaling Pathways

NFκB is a zinc-dependent transcription factor that forms dimers and stimulates the expression of cytokines, growth factors, and survival genes. Epigenetic modulation of NFκB is important for the expression of survival genes that mediate the resistance of HNSCC (and other tumor) to chemotherapy and radiotherapy [53,54,55]. During homeostasis, NFκB is held quiescent in the cytoplasm through an interaction with IκBα. Upon insult or stress, the IκB kinase (IKK) complex degrades the IκBα-NFκB complex by phosphorylating two critical serine residues at positions 32 and 36 on IκBα, which targets it for degradation by the 26S proteasome. The liberated NFκB then translocates to the nucleus, where it binds to specific DNA sequences that regulate the expression of immune responses, cell growth, and cell survival [54]. Further, NFκB signaling is increased following cisplatin exposure, leading to a chromatin modification that prevents histone acetylation and chromatin condensation, preventing cisplatin from binding to DNA and forming adducts, thereby inducing resistance [56].

Another well-investigated signaling molecule is TGF-β. The TGF-β signaling pathways regulate cellular functions critical for cell homeostasis, proliferation, differentiation, migration, and invasion [57,58]. TGF-β exerts its regulatory effects on cells via interactions with TGF-β surface receptors (TGFβRI, TGFβRII, and TGFβRIII). Both TGFβRI and TGFβRII are transmembrane serine/threonine kinases; however, TGFβRIII lacks intrinsic kinase activity and acts as a TGFβRII coreceptor. Multiple studies have uncovered TGF-β alterations in HNSCC, such as low expression of TGFβRII in poorly differentiated HNSCC [59,60,61,62,63,64]. A study of OSCC patient samples showed that TGFβRII mRNA expression was 64% lower in OSCC tissue than adjacent mucosa or normal oropharyngeal tissue [64]. This reduced expression of the TGFβRII protein was confirmed by immunohistochemical staining.

Another study showed that both TGFβRI and phosphorylated SMAD-2 (a downstream target) were undetectable or minimally expressed in 50% of the HNSCC cases examined [59]. Tumors lacking TGFβR expression have been reported to be more aggressive [61]. However, in a different study of HNSCC cells that were able to respond to TGF-β signaling, treatment with TGF-β1 increased in vitro sphere formation, the expression of markers of cancer stem cells (CSC), and the resistance to cisplatin [65]. TGF-β1 treatment of HNSCC CSC also increased the expression of p21 and the Nrf2 transcription factor, which regulates glutathione metabolism and other anti-oxidative pathways, ultimately leading to downstream resistance to cisplatin [66]. In support of this finding, HNSCC patients who failed to respond to combined radiation and cisplatin (CRT) therapy showed higher levels of TGF-β3 in the extracellular vesicle (EV)-fraction of their plasma compared to those who did respond [67]. TGF-β3 expression was also higher in cisplatin-resistant SCC cell lines than in sensitive cell lines. Deletion of TGF-β3 in these cells sensitized them to cisplatin and paclitaxel, which could be reversed with the addition of exogenous TGF-β3 [67]. These studies demonstrate that TGF-β signaling components may regulate the resistance of HNSCC to chemotherapy.

Paraoxonase-2, one of three members of the serum paraoxonase family, is a membrane protein expressed in various human tissues that has antioxidant and various other effects. It has been shown to be associated with several cancers [68,69]. Importantly, it also appears to regulate the sensitivity of OSCC cells to both radiation and chemotherapy [69,70].

### 3.5. EGFR-Targeting Therapies

The epidermal growth factor receptor (EGFR) is a member of the ErbB family of receptors. It is a transmembrane receptor with a cytoplasmic tyrosine kinase domain that can activate downstream signaling pathways such as Ras, Akt, and JAK-STAT [71]. Genetic alterations can lead to over-activation of EGFR signaling in HNSCC and other cancers. Alterations may include activating mutations, structural changes, or overexpression of the receptor or ligand. One mechanism by which the EGFR is overexpressed involves alterations in *EGFR* copy numbers (CN) or gene amplification, which is associated with a poor prognosis and worse clinical outcomes [72,73,74]. The EGFR is overexpressed in ~90% of HSCC cases. Increased EGFR levels correlate with decreased disease-free survival and OS, a shorter PFS, and increased local-regional relapse [75,76]. *EGFR* gene amplifications are found in 10–30% of HNSCC and reported in 49% of OSCC cases [74,77]. Aberrant EGFR signaling can stimulate cell proliferation, angiogenesis, invasion, and metastasis. EGFR expression is associated with resistance to key first-line therapies including cisplatin and radiation [71,78,79,80].

Following the initial discovery of its roles in cancer, amplification of the EGFR quickly became a target of interest for drug development and discovery. This led to the development of both monoclonal antibodies (e.g., cetuximab and panitumumab) and small molecule inhibitors of the EGFR tyrosine kinase domain (TKD) (e.g., gefitinib and erlotinib) [81,82]. As with many targeted therapies, a subset of patients have favorable responses, but many have innate resistance to EGFR-targeted therapies (e.g., due to mutant targets), and others will develop resistance during treatment (i.e., acquired resistance) [71,83].

One genetic variant of the EGFR, EGFRvIII, results in constitutive activation of the ATR pathway and chemoresistance and is expressed in 18–40% of HNSCC cases [74,84,85,86,87]. There has been some controversy over the detection of EGFRvIII in HNSCC samples. Some studies have been unable to detect this variant or detected it at very low levels in HNSCC samples, while others have detected EGFRvIII in as many as 75% of the cases they have examined (*n* = 108) [77,88,89]. These observations may be due to poor concordance among the assays used; it has also been reported that there is a poor correlation between the EGFRvIII mRNA and protein levels [74,90]. Although its expression frequency remains controversial, the EGFRvIII variant may be important in the resistance HNSCC to cancer therapies [85,91]. In human samples, higher levels of EGFRvIII correlated with a decreased PFS in a study of 47 patients [92].

The EGFRvIII variant has an in-frame deletion of exons 2–7 of the EGFR, resulting in a truncated protein that disrupts the extracellular ligand binding domain, leading to ligand-independent activation [87]. Overexpression of EGFRvIII in SCC cells increased in vitro cell proliferation, increased tumor xenograft growth in vivo, and decreased the responses of the tumors to cisplatin and cetuximab [85]. While cetuximab can bind EGFRvIII and cause receptor internalization, it does not significantly reduce the growth of HNSCC cells in vivo or in vitro [93,94]. Notably, EGFRvIII is more common in HPV- HNSCC cases, which are associated with a worse prognosis than HPV+ cases [74,87].

#### 3.5.1. AKT/P13K Pathway

The phosphoinositide 3-kinase/serine-threonine kinase (PI3K/AKT) pathway is activated by upstream signaling of extracellular receptors (i.e., EGFR and other G-protein coupled receptors) and previous studies have demonstrated that there is increased AKT signaling in cetuximab resistant cells [95]. One study identified that mutations in the extracellular domain of EGFR can cause epidermal growth factor (EGF)-independent signaling, leading to sustained AKT signaling and resistance to cetuximab [95,96]. Several pre-clinical methods have shown that dual targeting with cetuximab and AKT and or RTK inhibitors can have a synergistic effect on the resistant cell lines [95,97,98,99]. Although there have been studies indicating cetuximab resistance via this pathway, the mechanism for intrinsic and acquired resistance involving the AKT/PI3K pathway is mostly unknown. In certain cases of HNSCC, *PIK3CA* may be mutated, leading to overactivation of AKT/PI3K signaling and cetuximab resistance [98]. Another study relates Akt- and ERK1/2-driven cetuximab resistance to overexpression of hepatocyte growth factor [100]. Cruz-Duarte et al. discovered that phospholipase C gamma 1 (PLCγ1), an EGFR effector upstream of Akt signaling, can be used as a predictive biomarker for cetuximab response in colorectal cancer [101].

#### 3.5.2. MAPK Pathway

The mitogen-activated protein kinase (MAPK) pathway is also called the RAS/RAF/MEK/ERK pathway and is also related to HNSCC resistance. It was found that HNSCC cells were able to acquire resistance to five different inhibitors, including afatanib (a tyrosine kinase inhibitor) and cetuximab, via increased ERK1/2 expression and regulation of stem-cell markers [95,102]. A recent study published by Yang et al. examined the use of 7-Epitaxol in combination with cisplatin in vitro and in vivo and found that this paclitaxel metabolite reduces cell viability by inducing cell cycle arrest and apoptosis [103]. At the cell signaling level, the 7-Epitaxol reduces the phosphorylation of AKT, ERK1/2, and p38 to induce caspase-mediated apoptosis. It has also been shown that AXL upregulation in HNSCC cells and patient-derived xenografts (PDXs) is a possible mechanism of intrinsic resistance through the MAPK pathway [95]. Through treatment with a multi-targeting drug (cabozantinib), c-MET and AXL overexpression was inhibited in radioresistant and cisplatin-resistant HNSCC cells [104]. Cabozantinib, in combination with pembrolizumab, is currently in a phase II clinical trial for relapsed or metastatic HNSCC (NCT03468218). Based on a HNSCC tissue micro array analysis, AXL expression was associated with poorer overall and recurrence-free survival in human-papilloma-virus-negative (HPV-) HNSCC [105]. AXL, upstream of MAPK, signaling may be used as a prognostic biomarker.

#### 3.5.3. JAK/STAT Pathway

Another pathway with potential therapeutic targets is the Janus kinase (JAK)-signal transducer and activator of the transcription (STAT) pathway [95,106]. This pathway has been reported to be involved in both cetuximab and cisplatin resistance. Secretion of interleukin-6 (IL-6) and other cytokines activate the JAK/STAT pathway and promote stemness and survival cues in cancer cells and subsequent resistance to cisplatin treatment [107]. In cisplatin-resistant cells, STAT1- and STAT3-induced aldo–keto reductase family 1 member C1 (AKR1C1) was associated with cisplatin response and was a poor prognostic indicator in patient samples [108]. Cells with induced resistance to cetuximab overexpressed STAT3, and its knockdown resensitized cells [109]. Similar results were found by another group using cetuximab-resistant cells, and biopsies from cetuximab-resistant patients showed increased STAT3 activation [110]. Griso et al. state that STAT3, along with NF-κB activation, is more common in HPV- HNSCC [107]. A STAT3 targeting antisense oligonucleotide, Danvatirsen, is currently in phase II clinical trials for HNSCC and has been shown to enhance immune activation in combination with PD-L1 inhibitors (NCT05814666) [111].

## 4. Overcoming Therapeutic Resistance

The processes and molecules described above represent major targets for regulation or inhibition that might improve the patient outcomes. Approaches have been developed to overcome the resistance induced by each of the mechanisms. For example, to overcome resistance associated with epigenetic differences, pharmacological induction of chromatin acetylation via HDAC inhibitors such as trichostatin A or interfering with NFκB signaling have been shown to be promising interventions to reduce cisplatin resistance [20]. Although the data are relatively limited for HNSCC, the methylation status of cancer cells also seems to affect their radiosensitivity, suggesting that HDAC inhibitors may also lead to improved responses to radiation therapy [112,113].

A variety of NNMT inhibitors have been developed to treat numerous cancers [114]. The results have been dependent on the specific inhibitory molecule, cancer type, and type of study. However, overall, NNMT inhibition seems to have a variety of promising anti-cancer effects, including anti-proliferative, pro-apoptotic, and chemo- and radio-sensitizing effects [115]. No clinical trials have been performed yet for NNMT inhibitors, but numerous specific and multi-targeted agents have already been designed, and several natural product inhibitors have been identified that might be used as candidate treatments. Additionally, one of these natural products was shown to reverse the resistance of lung cancer to EGFR inhibition and might have similar effects in HNSCC [116].

Pharmacological inhibition of ATR using M6620 represents a possible approach to decrease DNA repair and increase the sensitivity to cisplatin, and this is currently being studied in phase 1 trials for HNSCC (NCT02567422) [117,118]. Inhibiting the ATR-CHEK1 pathway using siRNA or a small molecule inhibitor also sensitized a subset of OSCC cells (with 11q loss) to radiation [119]. Thus, targeting ATR appears to be a strategy that may be applicable for improving the responsiveness of HNSCC to various treatments.

Numerous approaches have been attempted to reinstate p53 activity in cancer cells [120]. These include approaches to (over)express wild-type p53, inhibit the pathways downstream of mutant p53, or to regulate the expression or activity of molecules that are normally regulated by wild-type p53. Unfortunately, despite the large number of approaches that have been explored, including several in human clinical trials, none have been approved for clinical use [121].

High-throughput drug screening initiatives have identified many compounds that inhibit the BCL-2 family of proteins in HNSCC [16,52,122,123]. Navitoclax (previously ABT-263) is an orally available agent that inhibits both BCL-2 and BCL-XL [52,122,123,124]. A recent in vitro study found that the effectiveness of navitoclax was limited as a monotherapy but that it demonstrated synergy when combined with cisplatin or radiation [15,122,125]. When it was combined with A-1210477, an MCL-1 inhibitor, there were synergistic effects that led to increased apoptosis in HPV-negative cells [52]. Venetoclax (ABT-199), another BCL-2 inhibitor, has also been shown to inhibit proliferation and induce apoptosis in a panel of oral squamous cell carcinoma cells. Mechanistically, venetoclax decreases the mitochondrial membrane potential, mitochondrial respiration, and overall ATP levels [126]. Importantly, these reports cumulatively suggest that targeting members of the BCL-2 family of proteins may represent a promising therapeutic approach for HNSCC. These findings also underscore the idea that targeting multiple molecules (e.g., all three anti-apoptotic BCL-2 family members) might be necessary to improve the clinical outcomes for patients by more effectively preventing or addressing resistance by preventing cells from using alternative or redundant pathways.

Several TGF-β signaling suppressors have been proposed for use in combination with chemotherapy, intended to either block TGF-β signaling or inhibit downstream SMAD proteins [57]. However, these combinations have not yet been explored for the clinical treatment of HNSCC. Similarly, while preliminary studies using different inhibitors (a Wnt inhibitor and antisense oligonucleotides) had promising results, no paraoxonase-2 inhibitor has been tested for cancer therapy in clinical trials [69,70].

Glutathione (GSH) metabolism may also play a role in chemotherapeutic resistance. GSH may be added to cisplatin by glutathione S-transferase (GST). Platinum–GSH conjugates are formed, which increases the excretion of cisplatin, diminishing its tumor uptake [12,127]. Low expression of GST has been associated with a higher rate of response to chemotherapy in HNSCC patients [128]. One GST isoform, GST-π, was shown to be amplified in HNSCC samples that were more resistant to cisplatin than those with lower copy numbers of the gene [129]. Targeting GST in cancer cells may thus be another therapeutic avenue by which to sensitize resistant tumor cells to chemotherapy, which will eventually need to be investigated in human patients.

Numerous studies have been performed to address resistance to EGFR inhibitors. It was shown that ABT-806, a humanized EGFR antibody, binds to EGFRvIII with higher affinity than cetuximab. In a preclinical model of HNSCC, ABT-806 reduced the growth of xenograft tumors expressing both EGFRvIII and wild-type EGFR, suggesting that it may provide a therapeutic option for HNSCC with EGFRvIII [94]. Alternatively, several therapeutics under development were designed to target both the EGFR and other members of the ErbB family (e.g., HER2 and HER3). It is expected that this may help to overcome resistance related to redundant or alternative signaling. Afatinib, a dual EGFR and HER2 inhibitor, led to increases in the response rates and PFS compared to methotrexate in patients with refractory or metastasis HNSCC when used as a single agent [96]. A companion study showed that this oral medication was well-tolerated, and patients adhered well to the prescribed regimen [130]. A current phase II trial is examining the combination of afatinib with cetuximab in patients with HNSCC who previously received a platinum therapy and/or an immune checkpoint inhibitor (NCT02979977). Patritumumab, which targets another ErbB family member (HER3), did not show any benefit over the standard cetuximab and platinum regimen when it was used in combination with cetuximab and platinum chemotherapy; however, it may be useful in different combinations [131].

Multi-target tyrosine kinase inhibitors (m-TKIs) have also been developed in an attempt to overcome the innate and acquired resistance that are common in many cancers [132]. Unfortunately, these therapeutics did not produce better patient outcomes, or their use was limited by severe toxicities (e.g., sunitinib (NCT00906360), vandetanib (NCT00459043), and dasatinib (NCT00507767) in the regimens and formulations tested [132,133]).

In addition to the major pathways of resistance discussed, there are also various other pathways and mechanisms that may enhance resistance. Potential therapeutics targeting many signaling pathways and molecules have been recommended to treat HNSCC, particularly recurrent and metastatic HNSCC (R/M HNSCC). Many of these are currently being explored in clinical trials (Table 1). These targets include immune system checkpoint molecules (e.g., PD-1 and CTLA-4), CDK4/6, PI3K, mTOR, PARP, and IAPs, among others [134,135,136]. 

Two immune checkpoint inhibitors, pembrolizumab and nivolumab, were recently approved for the treatment of HNSCC and OSCC [137,138,139]. Both agents are human antibodies targeted to programmed death-ligan 1 (PD-L1) that were designed to activate the immune response to kill cancer cells. Pembrolizumab is approved as a first-line therapy for R/M HNSCC in combination with platinum-based chemotherapy and fluorouracil or as a single-agent treatment for patients with PD-L1+ tumors [19]. Nivolumab is approved for the treatment of relapsed or metastatic HNSCC after the patient’s tumor has progressed on platinum therapy. A phase III trial of nivolumab reported that a subgroup of patients with R/M HNSCC whose tumors had progressed on platinum therapies responded to nivolumab, with a quarter of patients experiencing a reduction in tumor size [138]. Unfortunately, only about 20% of patients with HNSCC/OSCC typically respond to immune checkpoint inhibitors [118,140,141,142,143,144]. Of those who do respond, only a subset will have a durable response, while others will acquire resistance [140,141,142,143,144]. To diminish acquired resistance or bypass innate resistance, many studies and clinical trials are combining therapies in the hopes of producing a synergistic effect. For example, there are a number of ongoing trials exploring the combination of radiotherapy with these agents to determine whether this might lead to greater responses [11]. The combination of radiation therapy with immune checkpoint inhibitors is expected to result in increased tumor responses and possible tumor regression based on preclinical and preliminary clinical data [139].

In a phase Ib/II trial combining pembrolizumab with cetuximab, there was an objective response rate (ORR) of 45%, which is better than the previously reported response rates of these agents when they were administered alone [145]. Another phase 1b/II trial combined pembrolizumab with lenvatinib, a m-TKI that targets many growth factors receptors, RET, and c-KIT [146]. This trial showed an ORR of 36% in HSNCC patients. Many additional clinical trials using combinations of checkpoint inhibitors, either combined together or combined with standard-of-care therapies, are currently ongoing.

Early clinical trials (phase I or II) have identified many other promising targets for R/M HNSCC for use as single therapies or in combination with conventional treatments. Palbociclin, a CDK4/6 inhibitor, showed good efficacy against platinum- or cetuximab-resistant HPV- HNSCC when used in combination with cetuximab [147]. Buparlisib (BKM120), a pan-PI3K inhibitor, led to an increased PFS and OS when used in combination with cetuximab [148]. In patient-derived xenografts obtained from clinical trial participants, the combination of buparlisib and cetuximab was shown to synergistically induce apoptosis and reduce the tumor volume. Targeting PI3K may also sensitize tumors to radiation [149]. Treatment of xenograft tumors with LY294002, a PI3K inhibitor, was not effective alone, but led to synergistic anti-tumor effects when it was used in combination with radiation.

The mTOR complex 1 (mTORC1) is active downstream of PI3K [150,151]. Temsirolimus, a mTORC1 inhibitor, was not effective as a single agent, but was well tolerated when combined with cetuximab and may provide additional effects [151]. In support of this, a phase II clinical trial combining temsirolimus with carboplatin and paclitaxel resulted in a relatively high response rate in patients with R/M HNSCC [152]. A dual inhibitor of PI3K/mTOR, gedatolisib (previously PF-05212384), is currently being investigated in combination with a CDK4/6 inhibitor, palbociclib, in a phase I clinical trial including HNSCC patients (NCT03065062). Gedatolisib was shown to sensitize HNSCC cells to cetuximab and radiation in preclinical in vitro and in vivo models, and it is hoped that it will act additively or synergistically with these treatments in patients [153,154].

Poly-adenosine diphosphate-ribose polymerase (PARP) is an important enzyme that facilitates DNA repair in response to single strand breaks. PARP inhibitors appear to be promising cisplatin-sensitizers in a variety of cancers, including HNSCC [118,155]. Several clinical trials are being performed to elucidate the effects cisplatin in combination with PARP inhibitors such as olaparib or veliparib [155]. These agents may also be useful to improve the response to radiotherapy. An in vivo study of HNSCC suggested that PARP inhibitors can enhance the sensitivity of HNSCC tumor cell spheroids to radiation, leading to growth inhibition, although the extent of the growth inhibition was dependent on the specific inhibitor used and the type of HNSCC. Interestingly, the effects were stronger for HPV-negative oropharyngeal HNSCC than HPV-positive cells [156].

The inhibitors of apoptosis proteins (IAPs; XIAP, cIAP-1, and -2) regulate cell death and survival and are frequently overexpressed in HNSCC, particularly in HPV-negative tumors [157]. IAP inhibitors (including molecules mimicking the natural IAP inhibitor, SMAC) can increase apoptosis, and such agents have been shown to sensitize cancer cells to both chemotherapy and radiation [158]. Several preliminary clinical trials of these inhibitors have shown favorable safety profiles, and many trials are currently ongoing.

## 5. Predicting the Response to Treatment

As noted above, there are a variety of potential targets and markers of resistance that may be useful for predicting the response of HNSCC to different treatments. While patients are now routinely tested for their tumor’s HPV, EGFR, and PD-L1 status at most institutions, testing for other markers is often restricted to research or clinical trials due to the unclear clinical benefit and associated costs. Nevertheless, there is a trend toward more extensive and individualized testing, with many larger institutions performing tests ranging from pharmacogenomic studies to guide the selection of chemotherapy to liquid biopsies (focused primarily on circulating tumor cells) to genetic sequencing or artificial intelligence (AI)-guided risk stratification of biopsy samples to determine the prognosis before treatment is initiated [159,160,161].

Advanced imaging studies are also being used to evaluate the response of tumors and metastases to different treatments. For example, both positron emission tomography (PET) scanning and magnetic resonance imaging (MRI) can be used to monitor the response to chemotherapy and radiation [162,163]. Interestingly, imaging may also be useful for classifying tumors, as preliminary studies have shown that the features of HPV-positive and HPV-negative tumors differ, particularly in computed tomography images [164]. Imaging may also be used to predict the response to treatment through use of AI [161].

Although further research is needed to fine-tune their utility, other studies are examining the use of patient-derived xenograft or cell culture-based experiments to predict the response to therapy prior to the start of treatment [165,166]. These types of experiments may permit the testing of a variety of different agents and combinations of agents ex vivo, allowing for the most effective regimen to be selected for each patient or each tumor. It is expected that combining the results of these types of studies with sequencing data and machine learning/AI may yield a more individually tailored treatment that could result in better outcomes. It would also provide the opportunity to perform in silico testing of other agents (new classes of chemicals or repurposing existing approved drugs), leading to new treatment options [167,168,169].

## 6. Conclusions

The resistance of HNSCC to conventional treatments has driven the development and testing of new targeted therapies that can act upon multiple cellular pathways to target resistant cancer cells. Combinations of chemotherapeutic drugs, small molecular inhibitors, and antibody-based therapeutics, with or without radiation, may be necessary to overcome resistance. Phase I/II clinical trials have shown some promising results for dual or combination therapies against HNSCC. However, there are still many factors that need to be considered for the individual patients when selecting treatments, including their intrinsic resistance to the different treatments, the optimal combination of treatments to eradicate the tumor while minimizing adverse effects, and the identification of biomarkers to predict the response to and monitor the effects of treatment. Such improvements should be part of the standard of care in this era of personalized medicine. The use of computer-aided diagnostics and treatment planning will likely improve the outcomes of patients in the future.

## Figures and Tables

**Table 1:** Clinical trials for the treatment of recurrent or metastatic HNSCC.

Experimental Treatment	SOC Treatment Used in Combination	Phase of Trial	Target of Experimental Therapy	Identifier	Status
Elimusertib (BAY-1895344)	Pembrolizumab and Radiation	I	ATR	NCT04576091	Active; not recruitiing
Berzosertib (M6620)	Cisplatin/Radiation	I	ATR	NCT02567422	Active; not recruitiing
Cabozantinib	Pembrolizumab	II	AXL/MET/VEGFR2	NCT03468218	Active; not recruitiing
Palbociclib/Gedatolisib	—	I	CDK4/6, PI3K/mTOR	NCT03065062	Recruiting
NRC-2694-A	Paclitaxel	II	EGFR tyrosine kinase	NCT05283226	Recruiting
SI-B001	—	I	EGFR/HER3	NCT04603287	Recruiting
SI-B001	Platinum-based chemotherapy	II	EGFR/HER3	NCT05044897	Recruiting
Afatinib	Cetuximab	II	ErbB tyrosine kinase	NCT02979977	Recruiting
Vorinostat	Cisplatin/Radiation	II	HDAC	NCT05608369	Not yet recruiting
Vorinostat	Pembrolizumab	II	HDAC	NCT04357873	Active; not recruitiing
Abexinostat	Pembrolizumab	I	HDAC	NCT03590054	Completed
Xevinapant	Cetuximab	III	IAPs	NCT05930938	Recruiting
NT-I7	—	I	Interleukin-7 fusion protein	NCT04588038	Recruiting
NT219	Alone or with Cetuximab	I/II	IRS1/2 and STAT3	NCT04474470	Active; not recruitiing
Olaparib	Pembrolizumab and Carboplatin	II	PARP	NCT04643379	Active; not recruitiing
Nivolumab	Cisplatin/Radiation	III	PD-1	NCT03576417	Recruiting
Nivolumab	—	II	PD-1	NCT03355560	Active; not recruitiing
Pembrolizumab	Cisplatin or Carboplatin/Docetaxel	II	PD-1	NCT05726370	Recruiting
Nivolumab	Nab-Paclitaxel	II	PD-1	NCT04831320	Active; not recruitiing
Cemiplimab	Paclitaxel and Carboplatin	II	PD-1	NCT04862650	Recruiting
Nivolumab/Lirilumab	—	II	PD-1, Killer-cell immunoglobulin-like receptors	NCT03341936	Active; not recruitiing
Durvalumab/Decitabine	—	I/II	PD-1/DNA methyltransferases	NCT03019003	Active; not recruitiing
Duvelisib	Docetaxel	II	PI3K	NCT05057247	Active; not recruitiing
Buparlisib	Paclitaxel	III	PI3K	NCT04338399	Active; not recruitiing
Sorafenib Tosylate	Cisplatin/Paclitaxel	II	protein kinases (VEGFR, RAF, etc.)	NCT00494182	Active; not recruitiing
Pepinemab	Pembrolizumab	II	SEMA4D	NCT04815720	Recruiting
Sonidegib	Pembrolizumab	I	Smoothened	NCT04007744	Recruiting
Danvatirsen	Pembrolizumab	II	STAT3	NCT05814666	Recruiting
Ramucirumab	Pembrolizumab	II	VEGFR-2	NCT05980000	Recruiting
Adavosertib (AZD1775)	Cisplatin/Radiation	I	WEE-1 kinase	NCT02585973	Completed
Adavosertib	Cisplatin/Docetaxel	I	WEE-1 kinase	NCT02508246	Completed

ATR, ataxia telangiectasia and Rad3-related; CDK, cyclin-dependent kinase; EGFR, epidermal growth factor receptor; HDAC, histone deactylase; PI3K, phosphoinositide 3-kinase; PARP, Poly (ADP-ribose) polymerase; PD-1, programmed cell death protein 1; VEGFR, vascular endothelial growth factor receptor. Source: Clinicaltrials.gov, accessed on 1 December 2023.
